# Multiple Pituitary Adenomas: A Systematic Review

**DOI:** 10.3389/fendo.2016.00001

**Published:** 2016-02-01

**Authors:** Renata M. Budan, Carmen E. Georgescu

**Affiliations:** ^1^Department of Endocrinology, “Iuliu Hatieganu” University of Medicine and Pharmacy, Cluj-Napoca, Romania; ^2^Endocrinology Clinic, Cluj County Emergency Clinical Hospital, Cluj-Napoca, Romania

**Keywords:** double pituitary adenomas, multiple pituitary adenomas, Cushing’s disease, acromegaly, immunohistochemistry, magnetic resonance imaging, pituitary transcription factor

## Abstract

PubMed, Scopus, and Web of Science Core Collection databases were systematically searched for studies reporting synchronous double or multiple pituitary adenomas (MPA), a rare clinical condition, with a vague pathogenesis. Multiple adenomas of the pituitary gland are referred to as morphologically and/or immunocytochemically distinct tumors that are frequently small-sized and hormonally non-functional, to account for the low detection rate. There is no general agreement on how to classify MPA, various criteria, such as tumor contiguity, immunoreactivity, and clonality analysis are being used. Among the component tumors, prolactin (PRL)-immunopositive adenomas are highly prevalent, albeit mute in the majority of cases. The most frequent clinical presentation of MPA is Cushing’s syndrome, given the fact that in more than 50% of reported cases at least one lesion stains for adrenocorticotrophic hormone (ACTH). Plurihormonal hyperactivity may be diagnosed in a patient with MPA when more than one tumor is clinically active (e.g., ACTH and PRL) or in cases with at least one composite tumor (e.g., GH and PRL), to complicate the clinical scenario. Specific challenges associated with MPA include high surgical failure rates, enforcing second-look surgery in certain cases, and difficult preoperative neuroradiological imaging evaluation, with an overall sensitivity of only 25% for magnetic resonance imaging to detect distinct multiple tumors. Alternatively, minor pituitary imaging abnormalities may raise suspicion, as these are not uncommon. Postoperative immunohistochemistry is mandatory and in conjunction to electron microscopy scanning and testing for transcription factors (i.e., *Pit-1*, *T-pit*, and SF-1) accurately define and classify the distinct cytodifferentiation of MPA.

## Introduction

Up to 10–20% of people harbor a pituitary tumor, mostly in the form of a benign, sporadic, or familial (1), monoclonal, and slow-growing micro- or macroadenoma while aggressive or true malignant tumors constitute a rare finding (2). Pituitary adenomas provide up to 90% of the sellar and parasellar region masses (3), generally featuring a solitary tumor associating clinical traits attributed to pituitary hormones dysfunction and/or local mass effects. To a great extent, small pituitary adenomas have a “quiet” presentation and may behave as silent, asymptomatic tumors, in spite of positive immunohistochemistry (IHC), while true null-cell adenomas are infrequent (4).

A body of clinical case reports published over the past quarter of century point out to clinically relevant, concurrent double, or multiple adenomas of the hypophysis. Multiple pituitary adenomas (MPA) represent simultaneous, but otherwise morphologically and IHC distinct masses of the pituitary gland (5) and need to be distinguished from mixt, multihormone-secreting tumors as such term a single pituitary lesion expressing two or several hormones at IHC. Albeit rare in the clinical setting, MPA are increasingly suspected when there is an incidental finding upon preoperative magnetic resonance imaging (MRI) evaluation of the pituitary gland. As they may pose a challenging clinical scenario with regard to imaging localization and optimal first-choice approach, occasionally enforcing thorough surgical exploration of the pituitary gland to achieve complete removal of functional adenomatous tissue, MPA deserve attention as a distinctive pituitary pathology.

By screening the literature for isolated cases or case series reports, we completed a systematic review to gain insight into current knowledge concerning the diagnosis and management of MPA. Particularly, clinically relevant aspects were highlighted.

## Study Methodology

A systematic search of the literature was performed in United States National Library of Medicine PubMed, Scopus, and Web of Science Core Collection databases with “multiple pituitary adenoma/tumor,” “double pituitary adenoma/tumor,” “pituitary magnetic resonance imaging,” and “pituitary immunohistochemistry” as key words. All identified clinical cases or case series were evaluated to be included in the analysis. Of these, only reports recording the patients’ clinical data, endocrine evaluation, and surgery outcome with IHC exam were retained. Case reports presenting metachronous MPA (6) or in which multiple concurrent tumors were not clearly documented in spite of a high index of suspicion (7) were excluded. Clinical cases reported on two different occasions were considered only once (8, 9). Autopsy studies lacking clinical and endocrine documentation (10) were not included in the analysis but discussed throughout the manuscript. However, three cases (two bearing prolactinomas and one non-functional adenoma), in which distinct double pituitary adenoma was confirmed by MRI, were not subjected to surgery (11–13). Double pituitary masses of different origin (e.g., adenoma and craniopharyngioma) were not considered (14). Ultimately, 63 patients harboring 129 (60 double and three triple) synchronous pituitary adenomas fulfilled the study criteria.

## Prevalence of MPA: The Size of the Problem

### Autopsy Reports

Since the first report on multiple pituitary tumors by Kraus in 1914 (15), estimates provided by autopsy studies on the prevalence of incidentally detected MPA varied widely, ranging from 0.01 to 9% of the examined pituitary glands (10, 16–25). Furthermore, cases with double, triple, and even 10 multiple tumors within one pituitary gland were recognized (17). In a large retrospective review of more than 9000 autopsies samples from St. Michael’s Hospital records and the Mayo Clinic Tissue Registry, Kontogeorgeos et al. described 20 tumor-transformed pituitary glands harboring multiple adenomas (i.e., 16 double and four triple), in total 44 tumors; size was measured in 30 tumors, all of which were microadenomas, the majority measuring <3 mm (10). Consistently, subsequent studies showed that 55–99% of incidentally found adenomas in autopsy materials measure <3 mm (21–23). When Kontogeorgeos et al. restricted their study to 470 randomly autopsied pituitary glands, a prevalence of 0.9% MPA was found, in fact 8.9% of adenomatous glands exhibiting multiple tumors (10). As stated by the authors themselves, the global prevalence of pituitary adenomas in the work of Kontogeorgeos et al. (10.4%) was twofold to threefold lower compared to the reports based on the examination of serial sections of the gland (10). Indeed, single-section examination allowed detection of pituitary adenomas in 2.7–13% of evaluated glands, whereas with serial sectioning of the specimens, the incidence reached 8.4–27% (17, 21–23, 25–27).

Regarding MPA, the emphasis should be on their prevalence among adenomatous pituitaries, which clearly shows that MPA is not an infrequent pituitary pathology. Evaluation of serial 1 mm sections of autopsied pituitaries showed a MPA prevalence of 18.6 and 34.3% in the study of Costello (17) and Burrow (22), respectively. About 8.3% double tumors in a small series of 24 adenomatous glands were found by Tomita et al. (28), while more recently Buurman and Saeger reported 17 (5.4%) multiple tumors out of 316 affected pituitaries (25). Large autopsy series pointed to rates of MPA of 7–10.5% of tumoral glands (17, 21–24, 29), the majority of (≈90%) presenting as double adenomas and only rarely as three tumors (10, 25). Ectopic tumors (30) or combination forms, such as anterior pituitary adenoma and posterior pituitary granular cell myoblastoma (31), have been reported.

### Surgical Specimens

Prevalence data in surgical specimens is rather scarce, mostly originating from isolated case reports or specific patient databases (Table 1) (5, 29, 32–35).

**Table 1:** **MPA incidence from five large surgical series, with specifications regarding most frequent clinical presentation**.

Incidence of multiple pituitary adenomas (MPA) in five large surgical series					

Reference	No. of glands studied	Glands with multiple adenomas		Clinical presentation No. of patients	
		No.	Percent	Cushing’s disease	Acromegaly
Powers and Wilson (35)	600	1	0.16	–	–
Kontogeorgos et al. (5)	3000	11	0.3	2	6
Pantelia et al. (56)	332	1	0.3	1	–
Sano et al. (32)	450	6	1.3	–	6
Ratliff and Oldfield (29)	660	13	1.9	11	–
Kim et al. (33)	600	4	0.6	–	4
Magri et al. (34)	117	3	2.6	–	1
Koutourousiou et al. (8)	548	1	0.18	–	1

Overall, the reported prevalence of MPA according to surgical data varies from 0.37% detected by Kontogeorgeos et al. in an unselected series of 3000 cases of pituitary adenomas (5) to 1.3% observed by Sano in a surgical specimens review of 450 cases (32) and 2.6% by Magri in 117 unselected pituitary adenoma patients (34) but is highly likely to be underestimated. In a series of 600 operated patients referred for pituitary adenoma, Kim et al. reported four (0.67%) cases (33), while Ratliff documented distinct double pituitary tumors in 1.9% of resected specimens (29). Variables including the tumor size or the size and quality of the surgical specimen submitted to the pathological exam, particularly on second-look interventions, might have impact on the estimates. In a small series of Cushing’s disease patients, incidentally identified adenomas had an average maximum diameter of 3 ± 4 mm (median 2 mm) (29). Detection of a second tumor is more difficult in the presence of a macroadenoma (e.g., GH-producing tumors), often associated with compression of surrounding pituitary tissue, potentially masking a second tumor located within the compressed gland (29, 36). Furthermore, clinically non-active, intrasellar adenomas, lacking a tumor mass effect, even if confirmed by MRI might not represent an indication for surgery (13). Hence, the prevalence of MPA might be underestimated.

## Classification and Morphology: The Clue to the Unknown

Synchronous MPA are classified upon macroscopic aspect into *clearly distinct tumors*, recognized as such by neuroradiological imaging and/or surgery (37, 38), and *contiguous (collision) tumors*, which are resected as a single tumor and confirmed as MPA by the pathologist (9, 32, 34, 39, 40). An interpolated section of non-tumorous pituitary parenchyma should be distinctively recognized in individualized adenomas as a histological criterion. In the contiguous form, specific cytology, IHC and ultrastructure features, in addition to a well-delineated border between the tumors prove existence of different cell populations. In spite of that, discrete morphological differences between cells may also feature single pituitary adenomas (39); *vice versa*, similar patterns do not exclude distinct immunoreactivity. However, some authors strictly defined MPA based on the evidence of distinct tumors (32).

Evaluation of multiple adenomas in autopsy materials most commonly demonstrated immune-positivity for PRL (10, 28); however, tumors staining for PRL are rather mute in the setting of MPA (10). Apart from PRL-immunopositive tumors, the majority of incidentally discovered multiple tumors is represented by FSH-secreting and null cell adenomas.

In surgical samples, earlier studies reported GH-immunopositive tumors as being most prevalent (10, 12, 32). Systematically, analyzing the 63 MPA case reports with IHC data, we observed a 50.7% chance that at least one tumor is adrenocorticotrophic hormone (ACTH)-immunopositive, which is in accordance to Cushing’s disease as the most prevalent clinical presentation (Table 2). Silent cell PRL-secreting adenomas or functional prolactinomas coexist, most frequently, with either GH- or ACTH-secreting tumors (Table 3). When excluding MPA assigned to genetic forms (i.e., MEN-1 syndrome), double GH- and PRL-secreting tumors appear to be the most prevalent combination. Indeed, higher rates of mixt ACTH- and PRL-secreting adenomas are typically seen in patients harboring the MEN-1 mutation, otherwise they are regarded as a rather rare clinical finding. Nonetheless, in the setting of MPA, PRL-secreting tumors were the most common incidental lesion coexisting with ACTH-secreting adenomas (Table 2) (7, 29), which potentially points toward a genetic common background. However, this was not systematically investigated. Other reports indicated combinations of ACTH-producing tumors to gonadotrophic tumors (36, 41, 42).

**Table 2:** **Clinical presentation of MPA**.

No. of cases	Clinical presentation	No. (%)
63 patients	Cushing’s syndrome	24 (38.09)
	Acromegaly	22 (34.92)
	Cushing’s syndrome and acromegaly	3 (4.76)
	Hyperprolactinemia syndrome	8 (12.69)
	Thyrotoxicosis	1 (1.58)
	None	5 (7.93)

**Table 3:** **Classification of MPA according to size, macroscopic, and IHC pattern**.

	No. of analyzed cases	Tumor group	No. of cases/tumors (%)	Clonality	
				IC No. (%)	DC No. (%)
IHC	63	GH-PRL-TSH and LH-FSH	31 (49.2)	17 (54.83)	14 (45.16)
		ACTH and GH-PRL-TSH	30 (47.61)	2 (6.66)	28 (93.33)
		LH-FSH and ACTH	2 (3.17)	0	2 (100)
Size	23^a^ (49 adenomas)	Macroadenomas	5 (10.2)		
		Microadenomas	44 (89.79)		
Macroscopic aspect	49^b^	Contiguous	23 (46.93)		
		Clearly distinct	26 (53.06)		

*IHC, immunohistochemistry; IC, identical clonality; DC, different clonality*.

*^a^23 case reports (i.e., 49 pituitary adenomas) were identified in that tumor size was assessed by MRI/surgery or IHC evaluation*.

*^b^49 case reports in that information was available*.

Regarding the immunoreactivity, it resulted that 17.82% (23/129) adenomas exhibited a pluriendocrine, mixt secretory pattern. The most frequent combination immunoprofile was represented by mixt GH and PRL secretion, in fact, resembling the high incidence of composite GH-PRL secretion encountered in single pituitary adenomas. In a review of double pituitary adenomas, including tumors with pathological report albeit incomplete endocrine evaluation, Iacovazzo et al. identified coexistent ACTH- and PRL-secreting synchronous tumors in 33% of cases, closely followed by combined non-functional and GH-secreting pituitary adenomas (24%), GH- and PRL-secreting tumors (10%), and non-functional adenomas combined with either ACTH-secreting (7%) or PRL-secreting tumors (7%). In most of the cases, tumors staining for PRL were silent (7).

Based on tumor immunoreactivity profile and assigned clonality, MPA should be classified into three main patterns (9): (1) GH-PRL-TSH-secreting adenomas combined with FSH-LH-secreting tumors; (2) GH-PRL-TSH producing adenomas combined with ACTH-secreting tumors; and (3) ACTH-secreting adenomas combined with FSH-LH-producing tumors, with the first two subgroups being identified as the most prevalent ones (Table 3).

Specifically, each of the tumors can secrete one or several hormones; hence, making different combinations of pituitary adenomas possible.

## Pathogenetic Considerations in MPA

A number of genetic (e.g., oncogenes, tumor suppressor genes, and cell cycle abnormalities) or epigenetic (e.g., CpG dinucleotides methylation, expression of micro-RNAs and large non-coding-RNAs, and modification of histone tails) events appear to be involved in pituitary tumor genesis. However, limited information is available with regard to oncogenesis of multiple adenomas of the pituitary gland.

### The Multiple-Hit (Multicentricity) Theory

Coincidental monoclonal expansion of two distinct genetically mutated pituitary cell types could result in the development of synchronous tumors. Evidence of distinct tumor capsules at microdissection of surgical specimens, recognized even in the contiguous tumor phenotype (29), favors the concept of a multicentric origin of MPA.

### The Transdifferentiation Theory

Alternatively, cells of one already constituted pituitary adenoma could transdifferentiate into another cell type, with different morphologic and phenotypic characteristics including secretory features and local behavior.

Insight to the pathogenesis of MPA was gained by immunohistochemical localization of transcription factors, which allows classification of pituitary tumors according to the lineage of cell differentiation irrespective of the tumor hormone substance. Occasionally, genetic analysis for clonality has practical value as the demonstration of true lineage infidelity in the pathology specimen readily suggests multiple adenomas (40), although single plurihormonal adenomas exhibiting heterogeneous cell populations of different clonal origin were also recognized (43). As such, combination patterns in that tumors releasing secretory products consistent with multiple clonal proliferations were certified by genetic analysis in few cases of composite pituitary adenomas (43, 44). Transcription factors immunoprofiling revealed in two of three cases of double pituitary adenomas lineages of distinct origin, with *Pit-1* expression in PRL-secreting cells and the hormone-negative cell component expressing *SF-1* (34). Likewise, *Pit-1* was expressed in PRL-secreting or pluriendocrine and *T-pit* in ACTH-secreting adenoma, while double adenoma combining *T-pit* and *SF-1* expressing cells resulted in development of ACTH- and gonadotroph-cell adenomas, as shown by Jastania et al. (40). Double tumors with different clonality rather support the true multiple adenomas theory (45), whereas transdifferentiation might be more plausible in cases of MPA sharing immunoreactivity to any of the hormones secreted by one of the three different lineages into which pluripotent progenitor cells from the normal pituitary gland differentiate (42).

Pituitary tumor-expressed growth factors and hormone receptors may participate in tumor development through auto- or paracrine mechanisms (46), and this hypothesis could apply for MPA as well. The particular condition of GH-secreting adenomas, which release mitogenic GH and IGF-1 to stimulate development of a second tumor, is prototypical (5).

Genetic abnormalities might play key roles in tumor genesis in patients with MPA as underpinned by positivity for MEN-1 mutation in 4/63 (6.34%) MPA cases included here, which are about 2.3-fold more frequent than expected in adult patients with pituitary adenoma (47). In addition to that, MEN-1 gene mutation was also confirmed in one of the patients with hyperprolactinemia harboring two distinct lesions on pituitary MRI (11), not included in our analysis. Notwithstanding, specific testing for genetic causes related to pituitary tumors was not offered to all patients, which in addition to the small study population may impair data accuracy, thus requiring further studies. In MEN-1 syndrome, the prevalence of tumors with more than one lineage is significantly higher compared to non-MEN-1 cases, with ACTH- and PRL-producing tumors as the most prevalent combination (48). Furthermore, Kim et al. (33) were able to report two cases of familial-isolated pituitary double GH-secreting adenomas (FIDPA), unrelated to MEN-1.

Additionally, somatic mutations (e.g., mutation of Gs protein) extended to more than one pituitary adenoma might be involved in the process of multiple tumor formation and have been demonstrated, particularly in GH-producing adenoma. Transcriptional studies may reveal whether coexisting tumors originate from one cell type (33). About 40% of GH-secreting adenomas harbor a Gs protein mutation (49), implying this mutation has a determinant tumorigenic role (50). A somatic mutation in the ubiquitin-specific protease 8 (USP8) gene (51) was reported in about one-thirds to two-thirds of ACTH-secreting adenomas, which is the most prevalent functional tumor found in MPA. Nevertheless, none of the cases with USP8 mutations exhibited double or multiple adenomas.

## Clinical Management of MPA

Of all cases, 45/63 (71.42%) beard hormonally active tumors in our analysis; therefore, recognition of double or multiple pituitary tumors within one pituitary gland is prerequisite to avoid failure of surgery by missing the causative lesion (29, 41, 52, 53).

It still is a matter of debate, which type of endocrine dysfunction essentially unravels the disease in patients harboring MPA, some case series reports favoring acromegaly (54), whereas others argue for Cushing’s disease (7).

Of 63 fully documented clinical cases reported until now, 24 presented as Cushing’s syndrome (5, 29, 30, 56, 36, 41, 42, 54–58), closely followed by acromegaly with 22 cases (5, 9, 32, 33, 39, 41, 50, 53, 54, 58–63). Of note, synchronous ACTH- and GH-producing tumors were confirmed by IHC in seven patients; three of these cases expressed clinically as Cushing’s syndrome, one as acromegaly, and three were diagnosed with both hyperadrenalism and acromegaly (54, 58, 59) (Table 3). However, particularly incipient disease clinical features in acromegaly might be mild; according to Endocrine Society guidelines, IGF-1 screening in each patient with confirmed pituitary mass is recommended (64). Hyperprolactinemia in acromegaly patients points out to either mixt GH and PRL tumor release or pituitary stalk compression in the setting of suprasellar macroadenoma but raises also the possibility of two independent GH- and PRL-secreting adenomas. In agreement to this, Tolis et al. reported a patient with acromegaly and galactorrhea–amenorrhea syndrome caused by two separate adenomas, one producing GH and the other PRL (53). Furthermore, failure of dopamine agonists’ effect in prolactinoma may suggest double pituitary adenoma in certain cases (65).

Apparently, about 1.6–3.3% of Cushing’s disease patients exhibit double or multiple pituitary tumors (29). Three cases of double pituitary lesions were reported by Meij et al. in patients with Cushing’s disease (42). Additionally, two out of three patients with double pituitary tumors reported by McKelvie et al. presented ACTH-dependent hypercortisolemia (41). By screening a Cushing’s syndrome database, Ratliff and Oldfield found double pituitary tumors in 13 of 660 operated pituitaries, prospectively evaluated (29). Up to the moment, only two clinical cases featuring clearly separated pituitary adenomas, both ACTH-secreting, were reported (30, 43). Cushing’s disease may be subclinical (59) and easily missed (7, 66).

The source of ACTH hypersecretion in ACTH-immunoreactive forms of MPA is mainly eutopic due to hyperfunctional pituitary corticotroph adenoma cells; however, ACTH may also derive from ectopic corticotroph tumor cells located in the parasellar area, the neurohypohysis, or the pituitary stalk (30, 67). Corticotroph cell nodular hyperplasia has been suggested in a small number of patients with Cushing’s disease (68), including MPA (42, 55).

Lack of disease remission after effective transsphenoidal surgery in a patient with Cushing’s disease supports, in theory, the possibility of residual tumor or MPA in that the incidental non-functional tumor was removed. Nevertheless, clear distinction between persistent disease after unsuccessful surgery and disease recurrence should be made (69), as the latter is also increasingly reported in cured pituitary ACTH-secreting adenoma (70).

Higher rates of Cushing’s disease among case reports possibly reflect more aggressive management enforced by the need to cure adrenal hyperfunction. However, variability between studies is relatively high and the small numbers of cases significantly impairs accuracy.

### Imaging Detection of MPA

Multiple adenomas of the pituitary gland are traditionally known as difficult to be detected at preoperative imaging investigation (5, 29, 42, 54), the widely accepted reason being the small size of at least one of tumors, in several cases <3 mm. Older studies reported low accuracy for MRI diagnosis of MPA, such as in the case series of Ratliff, where none of the seven cases harboring clearly separated double pituitary tumors identified by surgery had been previously detected by MRI as such (29). Nevertheless, a detailed overview of all MPA clinical case reports clearly shows that MRI was able to detect two or even three (56) pituitary lesions in about 25% of cases. Except for the five ACTH-secreting adenomas in the series of Ratliff, where MRI was non-diagnostic (29), a finding which is in agreement to up to about 40% of cases of Cushing’s disease having normal MRI (71), a single tumor image or, alternatively, ill-defined abnormalities, such as an asymmetric, inhomogeneous appearance of the gland (55) or a global increase in the pituitary volume (30) were noted in most patients, which might prompt the neurosurgeon to careful exploration of the gland. Most challenging situations remain contiguous MPA, resulting in single tumor appearance, even on high-resolution MRI exam (34) or macroadenomas masking small coexisting tumors (36, 57). As true MPA are rare, false-positive findings of double or multiple tumors on pituitary MRI might be detected; thus, diagnosis is confirmed following pathological exam.

High-resolution MRI techniques need to be employed in hormonally active tumors with non-diagnostic imaging tests (54). Thin-sliced and dynamic MRI represents a readily accessible option to improve resolution and increasingly detect multiple lesions. Alternatively, 3.0-T MRI could detect pituitary microadenomas not identified by 1.5-T MRI imaging (72). On the other hand, the higher sensitivity attributed to the higher field strength in >3.0-T MRI devices may also increase the likelihood of false positive results, and images have to be interpreted cautiously. Use of spoiled gradient recalled acquisition (SPGR) sequences improves detection of ACTH-producing adenomas (73, 74). Recently, more advanced techniques, such as MET-PET/3.0-T MRI, showed higher sensitivity in the detection of ACTH-secreting pituitary microadenomas compared to traditional and dynamic MRI (75, 76).

### Surgery and Postoperative Evaluation

Confirmed microadenoma at initial pituitary exploration prompts to the careful inspection of the anterior and lateral surfaces of the gland for a second tumor (29, 52) as more than 50% of small tumors, particularly GH- and PRL-secreting adenomas, present on the surface of the gland (5). Exploratory pituitary surgery should be reserved only to cases with high suspicion or during second-look surgery as well as for ACTH-secreting tumors with normal MRI and non-diagnostic inferior petrosal sinus sampling (IPSS). Intraoperative pituitary imaging [iUltrasonography (iUS)/iMRI] seems be useful to assure complete tumor removal in certain cases. Residual tumor detection is facilitated with iMRI, particularly when located in the para- or suprasellar area (77), to achieve rates of resection of up to 96% in unselected series (78); however, the sensitivity of the method significantly decreases when remnant lesions are smaller than 3 mm (79). Few studies reported on the use of iUS to allow targeted resection of small, hyperechoic pituitary adenomas, even in MRI-negative cases, as in the typical condition of Cushing’s disease (80, 81), although limited experience is available. Immunohistochemistry allows the exclusion of false double pituitary lesions by detecting distinct immunochemical signatures in surgical specimens.

Isolated case reports evoke Cushing’s disease patients in whom the surgeon removed a PRL- or GH-secreting adenoma while the ACTH-secreting tumor was identified and resected at reoperation (29, 41). Apart from absent ACTH stain on IHC, lack of postsurgical hypoadrenalism is highly suggestive of tumor persistence in operated Cushing’s disease patients (57). Even if both tumors are correctly removed, unequivocal diagnosis of MPA is difficult when adenomas show identical immunoreactivity; in these cases, variability in cells size, conventional staining, and cytoplasm appearance (36, 55) or presence of a thin lamellae of compressed normal pituitary tissue separating the tumors might be of help. Electron microscopy scanning may provide additional information (e.g., degree of granularity, changes in Golgi apparatus, or presence or absence of fibrous bodies) and should be considered when available (32, 33, 39, 40, 54, 65). Furthermore, quantitation of *O*-methylguanine-DNA methyltransferase (MGMT) immunoexpression, the Ki-67 nuclear labeling index, or supplemental transcription factors (82) may show significant differences among tumors in patients suspected for multiple adenomas.

## Conclusion

Autopsy series indicate a prevalence of 7–10% MPA of all adenomatous pituitary glands, which is highly discordant to exceedingly rare clinical and/or operative reports (i.e., 1–1.5% of pituitary adenomas), thus suggesting the many cases of MPA that remain undiagnosed, since only rarely these multiple tumors are clinically relevant. Notwithstanding, double lesion images or equivalent abnormalities on high-resolution MRI scan are not rare in patients harboring MPA and should always point to careful surgical pituitary exploration, particularly in Cushing’s syndrome or acromegaly patients. However, definitive diagnosis should be concluded based upon pathological examinations. Persistence of endocrine disturbances after pituitary surgery leads to a high index of suspicion for MPA. Up to this moment, the etiopathogeny of MPA remains elusive, in spite of various theoretical concepts and higher prevalence of MEN-1 mutation among genotyped cases. Further, molecular analysis will provide new insights into the pathogenesis of pituitary adenomas and the mechanisms of multidirectional phenotypic differentiation.

## Author Contributions

RMB collected data and drafted the manuscript. CEG is responsible for the design of the work, contributed to data analysis, and wrote the review.

## Conflict of Interest Statement

The authors declare that the research was conducted in the absence of any commercial or financial relationships that could be construed as a potential conflict of interest.
